# Does Support of Policies to Sustain the Care Economy Differ by Political Affiliation or Caregiving Responsibility?

**DOI:** 10.1093/geroni/igaf122.1441

**Published:** 2025-12-31

**Authors:** Katherine Miller, Jennifer Wolff, Karen Shen, Sandro Galea, Catherine Ettman

**Affiliations:** Johns Hopkins Bloomberg School of Public Health, Baltimore, Maryland, United States; Johns Hopkins University - School of Public Health, Baltimore, Maryland, United States; Johns Hopkins University, Baltimore, Maryland, United States; Washington University in St. Louis, St. Louis, Missouri, United States; Johns Hopkins Bloomberg School of Public Health, Baltimore, Maryland, United States

## Abstract

Identifying effective and financially viable strategies to meet the care needs of individuals with impaired function is a key policy challenge for the United States. The 2022 National Strategy to Support Family Caregivers identifies a range of actions to support caregivers while family-oriented policies to promote the affordability of care gain increasing attention. Using a nationally representative survey of adults in 2024, we examine public perceptions of federal policies to support older adults, adults living with disabilities, and their family caregivers by political affiliation and caregiving status. We find one-fifth of U.S. adults (20%) report caregiving responsibilities for adult(s) with disability, with no significant difference across political affiliation. We find widespread support for policies to sustain the care economy across demographic characteristics, caregiver status, and political affiliation. Endorsement was highest for policies to make care in facilities (79.0%) and homes (75.4%) more affordable, expand eligibility for financial access to care (77.3%), and increase capacity of the paid caregiving workforce (78.3%) – and was lower for expansion of paid family leave (65.4%) and payment of family caregivers (61.2%). Endorsement by political affiliation was most similar for policies of making care more affordable and least similar for paid family leave. Political affiliation is a major driver of endorsement of policies to support the care economy that is stronger in magnitude than sociodemographic characteristics, e.g., gender or caregiving. Public support of the care economy is strong—across caregivers and non-caregivers and political affiliations, suggesting policy action is a promising priority with bipartisan support.

